# Associations of white matter hyperintensity with frailty, sarcopenia, and nutritional risk: an analysis of an acute ischemic stroke patient database

**DOI:** 10.3389/fmed.2025.1631923

**Published:** 2025-10-15

**Authors:** Takayoshi Akimoto, Chiharu Sugi, Kenta Tasaki, Natsuki Oshita, Naotoshi Natori, Tomotaka Mizoguchi, Masaki Ishihara, Makoto Hara, Hideto Nakajima

**Affiliations:** Division of Neurology, Department of Medicine, Nihon University School of Medicine, Tokyo, Japan

**Keywords:** deep white matter hyperintensity, periventricular hyperintensity, frailty, sarcopenia, nutritional risk, acute ischemic stroke

## Abstract

**Introduction:**

White matter hyperintensity (WMH), frailty, sarcopenia, and nutritional risk are prevalent and critical factors in geriatric care. This study aimed to investigate the associations between WMH and frailty, sarcopenia, and nutritional risk status using data derived from an acute ischemic stroke (AIS) hospitalization database.

**Methods:**

Images of brain magnetic resonance imaging were reviewed for patients aged ≥60 years who were hospitalized for AIS. WMH was classified into deep white matter hyperintensity (DWMH) and periventricular hyperintensity (PVH) and graded on a scale from 0 to 3 using the Fazekas scale. Frailty was assessed on the basis of the frailty screening index derived from the interview data. Sarcopenia was diagnosed based on grip strength measured on the nonparalyzed side and muscle mass assessed using bioelectrical impedance analysis. The nutritional risk status was evaluated using the Geriatric Nutritional Risk Index.

**Results:**

DWMH of grade ≥1 was observed in 92% of patients and PVH in 94%. Trend tests for ordinal scales indicated that DWMH and PVH were associated with older age, a higher female proportion, greater stroke severity, longer hospital stay, and lower grip strength and muscle mass. DWMH alone was associated with a higher pneumonia incidence during hospitalization. PVH alone was significantly associated with an increased prevalence of frailty, sarcopenia, and nutritional risk status. To further explore these associations, logistic regression analyses were performed with grade ≥2 PVH, frailty, and sarcopenia as outcome variables. The analyses identified age, male sex, PVH, and sarcopenia as independent predictors of frailty; nutritional risk and frailty as predictors of sarcopenia; and frailty as the sole predictor of PVH.

**Discussion:**

WMH is highly prevalent among older adult patients with AIS, with PVH particularly associated with frailty, sarcopenia, and nutritional risk status. These conditions appear to be interrelated, and the findings suggest that sarcopenia and PVH can contribute to frailty development.

## 1 Introduction

White matter hyperintensities (WMHs) are lesions frequently observed on brain magnetic resonance imaging (MRI) in older adults. The pathogenesis of WMH is believed to encompass several factors, including cerebral microarteriosclerosis, hypoperfusion and hypoxia, impaired cerebrospinal fluid drainage, and vascular inflammation (1, 2). Aging has been identified as a major risk factor for WMH (2, 3), and the presence and severity of WMH have been associated with an increased risk of various neurological and cognitive impairments (4–6). Evaluating WMH is crucial considering their relationship with significant clinical outcomes in older adults.

Frailty, another condition frequently observed in older adults, is associated with an increased risk of adverse health outcomes, including falls, hospitalization, and mortality (7). The criteria proposed by Fried et al. (8) stated that an individual is classified as frail when three or more of the following five components are present: unintentional weight loss (emaciation), muscle weakness (reduced grip strength), self-reported fatigue (low endurance and energy), decreased walking speed (slowed gait), and low physical activity levels. Regarding WMH, aging is a major risk factor for frailty, and its prevalence increases with advancing age (7, 8). Frailty can lead to serious consequences, contributing to several age-related health issues and diseases.

Sarcopenia is characterized by a decline in muscle strength, muscle mass, or muscle quality owing to progressive and systemic dysfunction of the skeletal muscles in older adults (9, 10). It has also been associated with falls, cognitive impairment, Alzheimer’s disease, hospitalization, and mortality (10, 11). Moreover, undernutrition and nutritional risk status have been demonstrated to be associated with frailty and sarcopenia (12–14).

Understanding the interrelationships among these age-related conditions—WMHs, frailty, sarcopenia, and nutritional risk—is essential for developing effective diagnostic and therapeutic approaches aimed at improving the health of older adults. This study aimed to investigate how WMH, frailty, sarcopenia, and nutritional risk interact with each other in a cohort of patients with acute ischemic stroke (AIS).

## 2 Materials and methods

### 2.1 Eligibility criteria for patient inclusion

Eligible patients were those aged ≥60 years who were hospitalized for AIS from August 2021 to December 2022 and for whom written informed consent could be obtained from the patient or his/her family. AIS was defined as a case wherein a lesion was identified on a brain MRI diffusion-weighted image taken following the onset of new neurological symptoms or wherein AIS was the most appropriate cause of the neurological symptoms (e.g., older adult–onset medial longitudinal fasciculus syndrome) spanning >24 h. AIS severity was assessed using the National Institutes of Health Stroke Scale (NIHSS) on admission. The NIHSS classifies neurological symptoms caused by stroke into 15 items; each category is scored from 0 to 2 or 3 points, and the total score is calculated (15). The score ranges from 0 to 40; higher scores indicate more severe stroke symptoms (15).

Patients’ medical histories were reviewed for hypertension, diabetes, dyslipidemia, stroke (ischemic and/or hemorrhagic), smoking, and alcohol use. Patients who had smoked for >1 year, smoking most days of the week, were considered to have a smoking history. Patients who drank alcohol most days of the week were considered to have a drinking history.

Blood data were referenced to that at the time of hospitalization. After obtaining informed consent, height, weight, muscle mass, grip strength on the nonparalyzed side, and frailty were evaluated. In addition, carotid ultrasonography was performed during hospitalization to assess internal carotid artery stenosis, with measurements of maximum intima–media thickness (IMT) and peak systolic velocity (PSV). A PSV exceeding 200 cm/s is considered indicative of significant stenosis, defined as ≥70% narrowing according to the criteria of the North American Symptomatic Carotid Endarterectomy Trial (16). Furthermore, the complications of pneumonia and urinary tract infection during hospitalization were evaluated. The outcome was evaluated using the length of hospital stay and the modified Rankin Scale (mRS) at discharge. The mRS is frequently employed for evaluating the motor function of patients with stroke and is rated on a scale of 0–6, with 0 representing no symptoms and 6 indicating mortality (17).

### 2.2 WMH evaluation

For the evaluation of WMH, brain MRI T2 fluid-attenuated inversion recovery (FLAIR) images were obtained using the following equipment: Ingenia 3.0 T [Philips, Netherlands; repetition time (TR) = 10,000 ms; echo time (TE) = 125 ms; inversion time (TI) = 2,700 ms; field of view = 290 × 192 mm; slice thickness = 5 mm; interslice gap = 1 mm] or Ingenia 1.5 T (Philips, Netherlands; TR = 11,000 ms; TE = 120 ms; TI = 2,800 ms; field of view = 240 × 153 mm; slice thickness = 5 mm; interslice gap = 1 mm). Based on the images, WMHs were categorized into grades 0–3 according to the Fazekas classification (18). Deep white matter hyperintensity (DWMH) was graded on a scale of 0–3 [0, absent; 1, punctate foci (maximum size < 3 mm); 2, beginning confluence of foci (size ≥ 3 mm); and 3, large confluent areas]. Similarly, periventricular hyperintensity (PVH) was graded on a scale of 0–3 (0, absent; 1, a “cap” or pencil-thin lining; 2, a smooth “halo”; and 3, irregular PVH extending into the deep white matter). Two neurologists performed the classification; in case of disagreement in the evaluation, the patient was classified into the appropriate grade by consensus.

### 2.3 Definition of frail

Frailty was diagnosed on the basis of the following items: shrinking, physical function, physical activity, and exhaustion (8). However, walking speed, which is frequently used as an evaluation item for physical function and physical activity, is affected by AIS. In this study, the “frailty screening index,” a method for evaluating frailty based on interviews, was used for diagnosing frailty (19). The frailty screening index asks patients five questions. If the patient answers “Yes” to the following three questions, 1 point is added: “Have you lost 2 kg or more in the past 6 months?” “Do you think you walk slower than before?” “In the past 2 weeks, have you felt tired without a reason?” Furthermore, 1 point is added when a patient answers “No” to the following two questions: “Do you go for a walk for your health at least once a week?” “Can you recall what happened 5 min ago?” A total of ≥3 points is defined as frailty (19). In this study, the question was prefaced with “Please tell me about your condition before you were hospitalized for this stroke.”

### 2.4 Definition of sarcopenia

Sarcopenia was assessed by muscle mass and nonparalytic side grip strength according to the Asian Working Group for Sarcopenia 2019 criteria (9). Muscle mass was assessed using bioelectrical impedance analysis (Inbody S10, Seoul, South Korea), and low muscle mass was considered when the muscle mass was <7.0 and <5.4 kg/m^2^ for males and females, respectively. Grip strength was assessed as low when it was <26 and <18 kg for males and females, respectively. Patients with both low muscle mass and low grip strength were diagnosed with sarcopenia (9).

### 2.5 Definition of nutritional risk

In this study, the Geriatric Nutritional Risk Index (GNRI) was employed for evaluating nutritional risk (20). The GNRI is a simple tool for assessing the nutrition-related risks of morbidity and mortality in hospitalized older adults, calculated using serum albumin levels and the ratio of the actual weight to the ideal weight. The GNRI is calculated using the following formula: GNRI = 14.89 × albumin (g/dL) + 41.7 × (weight/ideal body weight). According to the original study, four nutritional risk levels were defined as follows: major risk (GNRI < 82), moderate risk (82 ≤ GNRI < 92), low risk (92 ≤ GNRI ≤ 98), and normal (GNRI > 98) (20). In this study, the ideal body weight was calculated as the weight at a body mass index (BMI) of 22 kg/m^2^.

### 2.6 Statistical analysis

To investigate whether the variables demonstrated a monotonic increasing or decreasing trend with respect to the PVH and DWMH grades, the Jonckheere–Terpstra test was applied for continuous variables, and the Cochran–Armitage test was used for nominal scales. The presence or absence of frailty and sarcopenia was analyzed using Fisher’s exact test. For nominal scales. The Shapiro–Wilk test was applied to both ordinal and continuous variables. Variables with normal distributions were analyzed using Student’s *t*-test, whereas variables without normal distributions were analyzed using the Mann–Whitney *U* test. When analyzing GNRI nutritional risk as a continuous variable, it was substituted as follows: normal, 1; low risk, 2; moderate risk, 3; and major risk, 4. Spearman’s rank correlation coefficient was calculated for the correlation between PVH and DWMH grades. Furthermore, binomial logistic regression analysis using the forced entry method was performed to explore the associations between frailty, sarcopenia, and PVH. The dependent variable was set as either frailty, sarcopenia, or PVH of grade 2 or higher, while the other two variables (e.g., when frailty was the dependent variable, sarcopenia and PVH grade ≥2 were used as independent variables), along with age, sex (male: 1; female: 2), and GNRI, were included as independent variables. For the independent variables included in the model, variance inflation factors were calculated using linear regression. All values were less than 10, indicating no evidence of multicollinearity. Statistical analysis was performed using SPSS 22.0 (IBM Corp., Armonk, NY, United States), with *p* < 0.05 considered statistically significant. To assess statistical power, *post hoc* power analyses were conducted using G*Power 3.1 for each outcome variable in the logistic regression models, focusing on categorical independent variables that demonstrated statistically significant associations. The analyses were based on the observed odds ratios, an alpha error of 0.05, and the corresponding sample sizes.

## 3 Results

### 3.1 Baseline characteristics of the participants

During the observation period, 172 patients with AIS were hospitalized. Of these patients, 27 were aged <60 years, 33 did not consent to the study, 1 did not have an MRI, 1 had missing weight data, and 1 had AIS recurrence within the study period and were therefore excluded from the analysis. Finally, 109 patients (median age, 79 years; 59.6% male) were included in the analysis. As 13 patients who had difficulty with the interview were excluded, the assessment of frailty was performed on 96 patients. Sarcopenia was assessed in 100 patients, excluding nine patients whose grip strength or muscle mass was not measured. Carotid ultrasonography examinations were performed in 101 patients. A PSV exceeding 200 cm/s, indicative of significant stenosis, was observed in only one patient. In this study, maximum IMT was analyzed as an indicator of carotid artery stenosis.

### 3.2 Comparison between DWMH and PVH grades

Of the eligible patients, 9, 21, 35, and 44 exhibited DWMH grades 0, 1, 2, and 3, respectively, and 7, 59, 13, and 30 had PVH grades 0, 1, 2, and 3, respectively. The 4 × 4 table (Table 1) shows a comparison of the grades of the two WMHs. The correlation coefficient between the DWMH and PVH grades was 0.662 (*p* < 0.001).

**Table 1:** Number of patients according to deep white matter hyperintensity and periventricular hyperintensity grades.

		DWMH grade				
		0	1	2	3	Total
PVH grade	0	4	2	1	0	7
	1	4	18	26	11	59
	2	1	1	5	6	13
	3	0	0	3	27	30
	Total	9	21	35	44	109

DWMH, deep white matter hyperintensity; PVH, periventricular hyperintensity.

Spearman’s rank correlation coefficient: *p* < 0.001, *R* = 0.662.

### 3.3 Univariate ordinal analysis for DWMH grade

The DWMH grade was higher in older adult patients (*p* = 0.041) and lower in males (*p* = 0.008). Physical measurements revealed that the higher the grade, the lower the height (*p* = 0.002), grip strength (*p* = 0.004), and muscle mass (*p* = 0.013); however, no significant relationship with BMI or sarcopenia rate was observed (Table 2). Moreover, no significant differences in the total frailty screening index and the proportion of frail individuals were noted. Regarding stroke severity, a trend for higher NIHSS scores with higher grades was observed (*p* = 0.006). No significant differences in medical history (hypertension, diabetes, dyslipidemia, and stroke); smoking history; drinking history; albumin; creatinine; C-reactive protein; hemoglobin A1c; maximum IMT; GNRI; or GNRI nutritional risk were noted. Regarding complications during hospitalization, higher DWMH grades indicated a higher pneumonia incidence (*p* = 0.028); however, no significant association with the incidence of urinary tract infection was observed. Additionally, higher grades were associated with longer length of stay (*p* = 0.005) and higher mRS at discharge (*p* = 0.001).

**Table 2:** Univariate analysis of the deep white matter hyperintensity grade among the study participants.

	Total (*n* = 109)	Grade 0 (*n* = 9)	Grade 1 (*n* = 21)	Grade 2 (*n* = 35)	Grade 3 (*n* = 44)	*p*
Age (years)	79 (60–99)	72 (61–86)	78 (60–95)	80 (60–99)	81 (60–96)	0.041^a^
Sex (male)	65 (59.6%)	8 (88.9%)	14 (66.7%)	23 (65.7%)	20 (45.5%)	0.008^a^
Height (cm)	160 (135–180)	166 (153–175)	163 (135–173)	160 (138–180)	155 (139–177)	0.002^a^
Body weight (kg)	58.5 (34–82.3)	68.0 (51.1–78)	57.0 (34–72)	60.0 (38.8–82.3)	55.5 (35.2–81.5)	0.074
BMI (kg/m^2^)	23.1 (14.3–33.8)	24.1 (16.7–29.2)	22.9 (18.6–26.9)	22.9 (14.3–29.2)	23.4 (14.3–33.8)	0.750
Frailty screening index^b^	2 (0–4)	1 (0–4)	2 (0–4)	2 (0–4)	2 (0–4)	0.117
Frailty^b^	26 (27.1%)	2 (25%)	5 (23.8%)	7 (21.9%)	12 (34.3%)	0.395
Grip power (kg)^c^	20.5 (2–45)	28 (3–45)	22 (6–36)	21 (2–42)	17.5 (2–35)	0.004^a^
Muscle mass (kg/m^2^)^c^	6.4 (3.4–10.5)	7.0 (5.8–8.9)	6.4 (4.5–8.7)	6.5 (4.5–10.5)	6.2 (3.4–8.1)	0.013^a^
Sarcopenia^c^	47 (47%)	3 (33.3%)	8 (40%)	15 (44.1%)	21 (56.8%)	0.121
NIHSS	2 (0–30)	1 (0–4)	2 (0–18)	2 (0–7)	3 (0–30)	0.006^a^
Drinking history	74 (67.9%)	5 (55.6%)	13 (61.9%)	24 (68.6%)	32 (72.7%)	0.232
Smoking history	53 (48.6%)	7 (77.8%)	10 (47.6%)	18 (51.4%)	18 (40.9%)	0.093
Hypertension	68 (62.4%)	4 (44.4%)	13 (61.9%)	22 (62.9%)	29 (65.9%)	0.316
Diabetes mellitus	30 (27.5%)	4 (44.4%)	6 (28.6%)	9 (25.7%)	11 (25%)	0.329
Dyslipidemia	40 (36.7%)	3 (33.3%)	10 (47.6%)	8 (22.9%)	19 (43.2%)	0.810
Stroke	3 (2.8%)	0 (0%)	2 (1.8%)	0 (0%)	1 (0.9%)	0.867
Albumin (g/dL)	4.0 (2.6–5)	4.1 (3.1–4.8)	4.1 (2.7–4.7)	4.1 (3.5–4.8)	3.9 (2.6–5.0)	0.173
Creatinine (mg/dL)	0.86 (0.5–9.1)	0.80 (0.6–1.5)	0.81 (0.5–2.1)	0.88 (0.5–9.1)	0.86 (0.5–4.6)	0.689
CRP (mg/dL)	0.13 (0.1–10)	0.1 (0.1–4)	0.12 (0.1–10)	0.12 (0.1–3.9)	0.15 (0.1–8.2)	0.315
HbA1c (%)	6.0 (5.0–10.8)	5.9 (5.7–10.3)	6.0 (5.1–8.0)	6.0 (5.2–8.6)	5.9 (5–10.8)	0.381
Max IMT (mm)	2.6 (0.9–8.0)	2.7 (1.4–4.1)	2.5 (1.5–5.1)	2.8 (0.9–8.0)	2.4 (0.9–5.9)	0.268
GNRI	103.7 (76.4–129.5)	103.4 (77.8–123.9)	103.5 (76.4–115.2)	103.9 (88.1–123.7)	103.8 (79.2–129.5)	0.509
GNRI nutritional risk	1 (1–4)	1 (1–4)	1 (1–4)	1 (1–3)	1 (1–4)	0.177
Pneumonia	14 (12.8%)	0 (0%)	1 (4.8%)	4 (11.4%)	9 (20.5%)	0.028^a^
UTI	18 (16.5%)	1 (11.1%)	4 (19%)	3 (8.6%)	10 (22.7%)	0.394
Length of hospital stay (days)	19 (3–68)	16 (7–25)	16 (11–52)	16 (5–34)	22 (3–68)	0.005^a^
mRS at discharge	3 (0–6)	2 (0–5)	1 (0–5)	2 (0–6)	4 (0–6)	0.001^a^

The numbers indicate the number of cases (%) or the median (minimum–maximum). BMI, body mass index; CRP, C-reactive protein; GNRI, Geriatric Nutritional Risk Index; HbA1c, hemoglobin A1c; Max IMT, maximum intima-media thickness; mRS, modified Rankin Scale; NIHSS, National Institutes of Health Stroke Scale; UTI, urinary tract infection.

a *p* < 0.05.

b Evaluated in 96 cases.

c Evaluated in 100 cases.

### 3.4 Univariate ordinal analysis for PVH grade

Similar to DWMH (Table 3), higher PVH grades were associated with older patients (*p* < 0.001), higher proportion of female patients (*p* = 0.035), higher NIHSS scores (*p* < 0.001), lower height (*p* = 0.003), lower grip strength (*p* = 0.001), and lower muscle mass (*p* < 0.001). Conversely, unlike DWMH, significant differences in body weight (*p* < 0.001), BMI (*p* = 0.017), total frailty screening index (*p* < 0.001), proportion of frailty (*p* = 0.003), and proportion of sarcopenia (*p* = 0.003) were observed. No significant differences in the rates of hypertension, diabetes, dyslipidemia, stroke or smoking history were noted. The higher PVH-grade group demonstrated lower alcohol consumption rates (*p* = 0.049). Regarding blood data and nutritional risk indicators, higher PVH grades were associated with lower albumin levels (*p* = 0.027) and GNRI (*p* = 0.004) as well as higher GNRI nutritional risk grade (*p* = 0.005). No significant differences were observed in maximum IMT among the PVH grade groups. Regarding the course of the patients following hospitalization, no significant difference in pneumonia and urinary tract infection complication rates was noted; however, the length of stay and mRS at discharge were both high (all *p* < 0.001).

**Table 3:** Univariate analysis of the periventricular hyperintensity grade among the study participants.

	Grade 0 (*n* = 7)	Grade 1 (*n* = 59)	Grade 2 (*n* = 13)	Grade 3 (*n* = 30)	*p*-value
Age (years)	67 (60–72)	78 (60–99)	85 (60–92)	81.5 (61–96)	<0.001^a^
Sex (male)	6 (85.7%)	37 (62.7%)	9 (69.2%)	13 (43.3%)	0.035^a^
Height (cm)	165 (148–173)	162 (135–180)	159 (143–175)	154 (140–170)	0.003^a^
Body weight (kg)	69.7 (57.0–74.0)	59.0 (34.0–82.3)	57.0 (49.0–75.8)	51.55 (35.2–75.4)	<0.001^a^
BMI (kg/m^2^)	24.6 (21.0–28.8)	23.5 (14.3–32.7)	23.5 (16.7–30.9)	21.7 (14.3–33.8)	0.017^a^
Frailty screening index^b^	1 (0–2)	2 (0–4)	2 (1–3)	2 (1–4)	<0.001^a^
Frailty^b^	0 (0%)	12 (21.1%)	3 (33.3%)	11 (47.8%)	0.003^a^
Grip power (kg)^c^	35 (18–38)	22 (2–45)	20 (3–31)	17 (2–40)	0.001^a^
Muscle mass (kg/m^2^)^c^	7.4 (6.0–8.1)	6.7 (4.5–10.5)	6.3 (5.0–8.1)	5.7 (3.4–7.8)	<0.001^a^
Sarcopenia^c^	0 (0%)	25 (43.1%)	5 (50%)	17 (68%)	0.003^a^
NIHSS	0 (0–6)	2 (0–18)	1 (0–26)	3.5 (1–30)	<0.001^a^
Drinking history	4 (57.1%)	22 (37.3%)	2 (15.4%)	7 (23.3%)	0.049^a^
Smoking history	5 (71.4%)	30 (50.8%)	6 (46.2%)	12 (40%)	0.156
Hypertension	3 (42.9%)	40 (67.8%)	8 (61.5%)	17 (56.7%)	0.653
Diabetes mellitus	4 (57.1%)	16 (27.1%)	2 (15.4%)	8 (26.7%)	0.351
Dyslipidemia	3 (42.9%)	25 (42.4%)	4 (30.8%)	8 (26.7%)	0.134
Stroke	1 (0.9%)	0 (0%)	1 (0.9%)	1 (0.9%)	0.867
Albumin (g/dL)	4.2 (3.7–4.8)	4.1 (2.7–5.0)	4 (2.6–4.4)	3.9 (3.0–4.8)	0.027^a^
Creatinine (mg/dL)	0.8 (0.7–1.0)	0.86 (0.5–9.1)	0.8 (0.6–2.1)	0.86 (0.5–4.6)	0.945
CRP (mg/dL)	0.1 (0.1–0.5)	0.12 (0.1–10)	0.26 (0.1–4.4)	0.12 (0.1–8.2)	0.326
HbA1c (%)	6.3 (5.8–10.3)	6 (5.1–8.0)	6.1 (5.5–8.6)	5.9 (5.0–10.8)	0.195
Max IMT (mm)	1.8 (1.1–4)	2.6 (0.9–8.0)	2.9 (0.9–5.9)	2.4 (0.9–5.2)	0.524
GNRI	109.7 (102.4–120.1)	104 (76.4–127.5)	105.5 (77.8–112.7)	99.4 (79.2–129.5)	0.004^a^
GNRI nutritional risk	1 (1–1)	1 (1–4)	1 (1–4)	1 (1–4)	0.005^a^
Pneumonia	0 (0%)	6 (10.2%)	2 (15.4%)	6 (20%)	0.099
UTI	0 (0%)	8 (13.6%)	2 (15.4%)	8 (26.7%)	0.056
Length of hospital stay (days)	11 (7–16)	17 (5–68)	21 (3–46)	22 (10–54)	<0.001^a^
mRS at discharge	1 (0–2)	2 (0–6)	2 (0–6)	3.5 (0–5)	<0.001^a^

The numbers indicate the number of cases (%) or the median (minimum–maximum). BMI, body mass index; CRP, C-reactive protein; GNRI, Geriatric Nutritional Risk Index; HbA1c, hemoglobin A1c; Max IMT, maximum intima–media thickness; mRS, modified Rankin Scale; NIHSS, National Institutes of Health Stroke Scale; UTI, urinary tract infection.

a *p* < 0.05.

b Evaluated in 96 cases.

c Evaluated in 100 cases.

### 3.5 Association between frailty and sarcopenia in AIS

Frailty was assessed in 96 patients, and 26 (27.1%) were diagnosed as frail on the basis of a frailty screening index of ≥3. Sarcopenia was observed in 47 of 100 patients (47%). A univariate analysis was conducted by stratifying patients according to the presence or absence of frailty and sarcopenia. The detailed results of this analysis are presented in Supplementary Tables 1, 2. Among patients with AIS, those with frailty exhibited a significantly higher sarcopenia prevalence (*p* = 0.001). The following were the common characteristics among patients with AIS having frailty and sarcopenia: older age (frail, *p* = 0.022; sarcopenic, *p* < 0.001), lower grip strength (*p* = 0.030 and *p* < 0.001), higher PVH grade (both *p* = 0.003), higher NIHSS score at admission (*p* = 0.008 and *p* = 0.001), longer hospital stay (*p* = 0.024 and *p* = 0.010), and higher mRS score at discharge (*p* = 0.010 and *p* < 0.001). Frailty was more common in males (*p* = 0.011) and was associated with a higher pneumonia incidence during hospitalization (*p* = 0.044). Patients with sarcopenia showed lower body weight, BMI, and muscle mass (all *p* < 0.001); lower albumin levels (*p* = 0.002); and lower GNRI but higher GNRI nutritional risk (both *p* < 0.001).

### 3.6 Multivariate analysis with frailty, sarcopenia, and PVH as dependent variables

**Figure 1:**
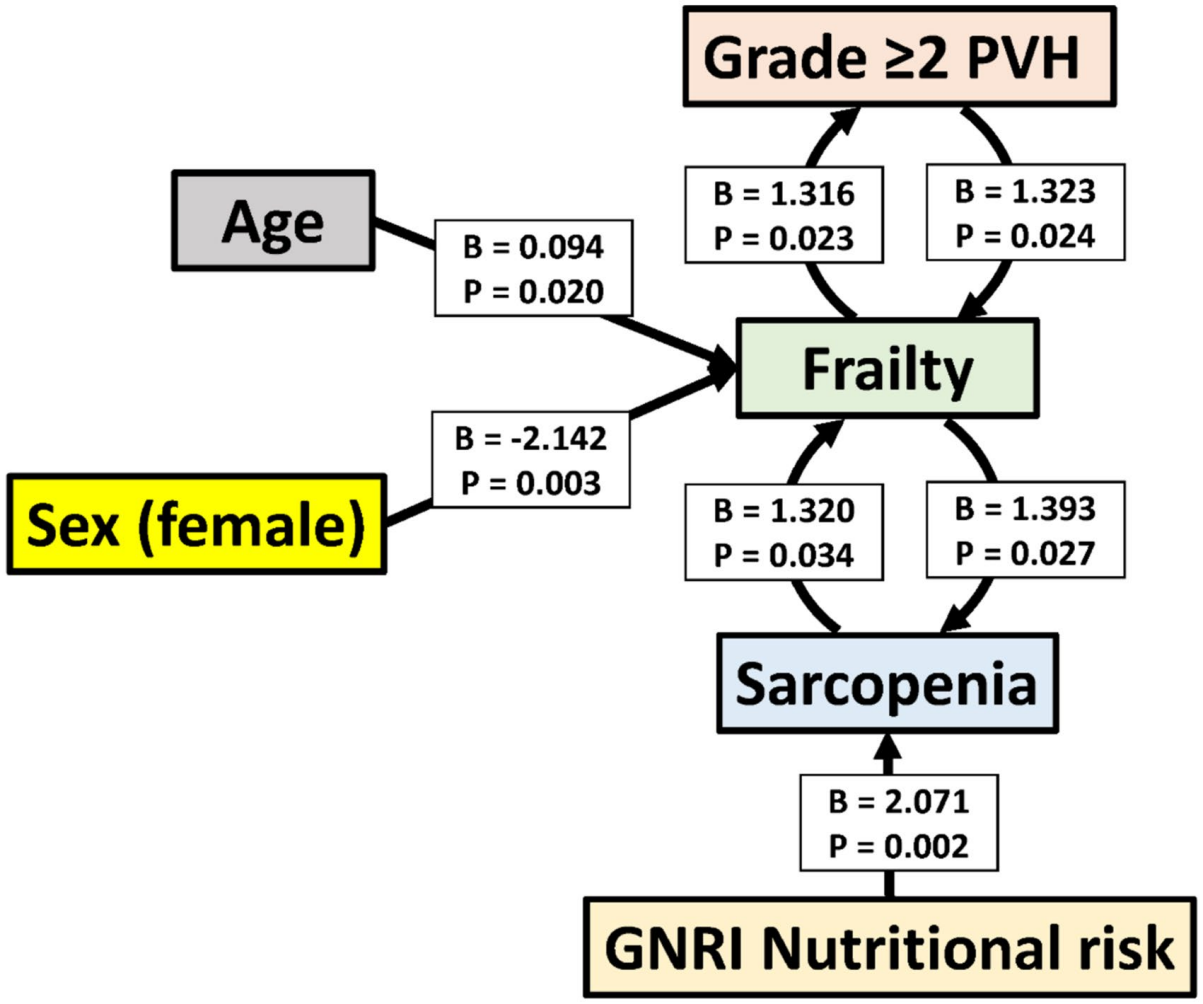
Associations among frailty, sarcopenia, grade ≥2 periventricular hyperintensity, and nutritional risk based on multivariate analysis. The figure includes only the variables with statistically significant associations in multivariate analysis. Arrows point from independent to dependent variables. B, unstandardized regression coefficient; CI, confidence interval; GNRI, Geriatric Nutritional Risk Index; PVH, periventricular hyperintensity.

In the ordinal logistic regression with the PVH grade as the dependent variable, the *p*-values for all of the threshold values were ≥0.05 (data not shown). Therefore, the analysis was conducted after converting the data to binary data, that is, whether the PVH grade was ≥2 or not (Tables 4–6). Frailty was significantly associated with age [*p* = 0.020; odds ratio (OR), 1.098; 95% confidence interval (CI), 1.015–1.188], male sex (sex was coded as male: 1 and female: 2; *p* = 0.003; OR, 0.117; 95% CI, 0.029–0.475), a PVH grade of ≥2 (*p* = 0.024; OR, 3.753; 95% CI, 1.186–11.879), and sarcopenia (*p* = 0.034; OR, 3.742; 95% CI, 1.106–12.655). In the analysis using sarcopenia as the dependent variable, GNRI nutritional risk (*p* = 0.002; OR, 7.937; 95% CI, 2.172–28.996) and frailty (*p* = 0.027; OR, 4.026; 95% CI, 1.173–13.823) were identified as significant factors. When a PVH grade of ≥2 was set as the dependent variable, frailty was the only significant predictor (*p* = 0.023; OR, 3.728; 95% CI, 1.195–11.628). The *post hoc* power analysis revealed that all relevant models demonstrated sufficient statistical power, with calculated power exceeding 0.9 in each case. Specifically, this included models with frailty as the dependent variable and sex, PVH grade, or sarcopenia as independent variables; sarcopenia as the dependent variable and frailty as the independent variable; and PVH grade as the dependent variable and frailty as the independent variable. Figure 1 illustrates the associations among frailty, sarcopenia, grade ≥2 PVH, and nutritional risk based on the results of multivariate analysis.

**Table 4:** Multivariate logistic regression analyses using frailty as the dependent variable.

Independent variables	B	SE	*p*-value	OR (95% CI)
Age	0.094	0.040	0.020^a^	1.098 (1.015–1.188)
Sex (female)	−2.142	0.713	0.003^a^	0.117 (0.029–0.475)
Grade ≥2 PVH	1.323	0.588	0.024^a^	3.753 (1.186–11.879)
GNRI nutritional risk	−0.107	0.507	0.834	0.899 (0.333–2.43)
Sarcopenia	1.320	0.622	0.034^a^	3.742 (1.106–12.655)

B, unstandardized regression coefficient; CI, confidence interval; GNRI, Geriatric Nutritional Risk Index; OR, odds ratio; SE, standard error; PVH, periventricular hyperintensity. *p*-value of the Hosmer–Lemeshow test: 0.441.

a *p <* 0.05.

**Table 5:** Multivariate logistic regression analyses using sarcopenia as the dependent variable.

Independent variables	B	SE	*p*-value	OR (95% CI)
Age	0.056	0.036	0.122	1.058 (0.985–1.135)
Sex (female)	−0.052	0.583	0.929	0.949 (0.303–2.978)
Grade ≥2 PVH	0.287	0.560	0.608	1.333 (0.445–3.995)
GNRI nutritional risk	2.071	0.661	0.002^a^	7.937 (2.172–28.996)
Frailty	1.393	0.629	0.027^a^	4.026 (1.173–13.823)

B, unstandardized regression coefficient; CI, confidence interval; GNRI, Geriatric Nutritional Risk Index; OR, odds ratio; SE, standard error; PVH, periventricular hyperintensity. *p*-value of the Hosmer–Lemeshow test: 0.977.

a *p* < 0.05.

**Table 6:** Multivariate logistic regression analyses using grade ≥2 PVH as the dependent variable.

Independent variables	B	SE	*p*-value	OR (95% CI)
Age	0.038	0.034	0.261	1.039 (0.972–1.111)
Sex (female)	0.588	0.540	0.277	1.8 (0.624–5.191)
GNRI nutritional risk	−0.131	0.392	0.738	0.877 (0.407–1.892)
Frailty	1.316	0.580	0.023^a^	3.728 (1.195–11.628)
Sarcopenia	0.348	0.540	0.520	1.416 (0.491–4.08)

B, unstandardized regression coefficient; CI, confidence interval; GNRI, Geriatric Nutritional Risk Index; OR, odds ratio; SE, standard error; PVH, periventricular hyperintensity. *p*-value of the Hosmer–Lemeshow test: 0.454.

a *p* < 0.05.

## 4 Discussion

This study investigated whether the DWMH and PVH grades were associated with frailty, sarcopenia, and nutritional risk using a database of patients admitted with AIS. First, univariate analyses revealed that higher DWMH and PVH grades were associated with older age, a higher proportion of females, lower grip strength and muscle mass, higher NIHSS scores at admission, higher mRS scores at discharge, and longer hospital stays. Notably, DWMH grade was uniquely associated with pneumonia incidence, whereas PVH grade was specifically associated with BMI, frailty and sarcopenia prevalence, and nutritional risk as assessed using the GNRI. Subsequently, multivariate analyses were conducted to clarify the interrelationships among frailty, sarcopenia, and PVH. Frailty was independently associated with advanced age, male sex, grade ≥2 PVH, and sarcopenia. Sarcopenia, in turn, was associated with frailty and nutritional risk, whereas grade ≥2 PVH was mainly associated with frailty.

The cerebrum is divided into gray and white matter. The gray matter, which covers the cerebral hemispheres as the cerebral cortex, is mainly composed of cell bodies, axons, and dendrites of neurons; it is involved in higher brain functions, including movement initiation, sensory perception, memory, and decision-making. The white matter of the cerebrum exists within the gray matter and comprises axons covered in myelin sheaths, which connect the cerebral hemispheres on the same or opposite side (21, 22). The brain is supplied by two internal carotid arteries and two vertebral arteries; the internal carotid arteries supply the anterior cerebral artery (ACA) and middle cerebral artery (MCA), while the vertebral arteries supply the posterior cerebral artery (PCA) (21, 22). After branching off the perforating arterioles, the three cerebral arteries reach the surface of the brain, where they form a network of arteries on the pia mater of the subarachnoid space (23). Owing to these anatomical characteristics, a watershed area exists between the border regions of the ACA, MCA, and PCA (24), as well as around the penetrating and perforating arterioles (23, 25, 26). Changes in the blood supply to these watershed areas due to arteriosclerosis lead to necrosis, cavitation, and rarefaction caused by local ischemia, causing WMH development (27, 28). WMHs are associated with several conditions and risk factors contributing to arteriosclerosis, including aging (29, 30), hypertension (3, 29), diabetes, high BMI (31), and chronic kidney disease (32). Furthermore, WMH is associated with impaired frontal lobe function, semantic memory (5), executive function, and attention (33); cognitive decline in patients with hypertension (4); mild cognitive impairment (34); Parkinson’s disease (5); Alzheimer’s disease; and aphasia (31). WMH is a risk factor for the postural instability and gait disorder motor phenotype (35).

WMHs appear as high-signal areas on FLAIR and T2-weighted MRI images. Lesions around the anterior and posterior horns of the lateral ventricles that extend upward are PVHs, whereas patchy lesions extending from the subcortical white matter to the centrum semiovale are DWMHs (18). The reported prevalence of DWMH and PVH varies widely, ranging from 39 to 96%, likely due to differences in MRI parameters, age distribution, or underlying diseases (36–40). Recent studies of patients with AIS have reported that the prevalence of grade ≥2 DWMH and PVH is approximately 60% (37, 38). In the present study, 72% of the patients had grade ≥2 DWMH, whereas 39% had grade ≥2 PVH.

The results of this study demonstrated that higher DWMH and PVH grades were associated with older age, a higher proportion of females, increased stroke severity on admission, longer hospital stays, and greater neurological disability at discharge. Being female is a significant risk factor for DWMH and PVH prevalence (41). Conversely, patients with higher PVH grades exhibited lower BMI values, higher frailty screening index scores, higher frailty and sarcopenia prevalence, lower albumin levels, and nutritional risk. These findings suggest that PVH might have a stronger impact on malnutrition, nutritional risk, and frailty than DWMH; this may be due to the differences in the pathophysiology underlying PVH and DWMH development. PVH is believed to be associated not only with atherosclerosis but also with impaired interstitial fluid drainage caused by medullary vein collagenization (2). Such venous drainage disorders may impair the glymphatic system, a brain waste clearance mechanism, potentially resulting in the accumulation of pathological proteins, including β-synuclein, β-amyloid, and α-synuclein (42). Additionally, PVH is associated with significantly greater axonal loss, astrocytic burden, microglial density, and oligodendrocyte loss than DWMH (43), suggesting that PVH can indicate more severe tissue damage than DWMH. Another possible explanation involves the anatomical characteristics of the regions where PVH develops. The frontal lobe contains the following three major groups of nerve fibers: projection, commissural, and association fibers. Association fibers are further classified into two groups—those within the frontal lobe and those connecting the frontal lobe to other brain regions (44, 45). In humans, such frontal lobe networks, extending both within and beyond the frontal lobe, are believed to account for 66% of the entire cerebral cortex (44). Moreover, DWMH may be sporadically observed in lobes other than the frontal lobe, predominantly affecting short cortical connections composed of arcuate U-fibers densely located immediately beneath the gray matter (46). In contrast, PVH is characterized by lesions resembling caps or halos around the lateral ventricles’ anterior horns and influences longer association fibers connecting distant cortical regions (46). PVH has been demonstrated to have a closer association with frontal lobe dysfunction, including attention, executive function, and cognitive performance deficits (4, 34). PVHs may contribute to such higher brain dysfunction, thereby leading to slower walking speed, reduced physical activity, and executive impairment in tasks including nutritional intake, ultimately promoting frailty, sarcopenia, and heightened nutritional risk.

Multivariate analysis was conducted to investigate the interrelationships among frailty, sarcopenia, and PVH. Sarcopenia and frailty, as well as PVH and frailty, were mutually significant explanatory variables. Frailty was associated with older age and male sex, whereas sarcopenia was associated with nutritional risk. However, PVH was not independently associated with sarcopenia. These results support the following hypotheses: aging and male sex increase the likelihood of frailty; frailty, in turn, may reciprocally exacerbate sarcopenia and PVH; and nutritional risk is a contributing factor to sarcopenia. A study of hospitalized older adults identified WMH as a potential factor linking sarcopenia and cognitive impairment (6). Furthermore, physical frailty is believed to contribute to the secondary development of social frailty (47), suggesting that conditions, including frailty, sarcopenia, and PVH, can subsequently lead to the development of other health issues.

This study has several limitations. As it was conducted at a single center, the sample size was limited. Additionally, two types of MRI scanners were used, including 1.5 T and 3.0 T. Previous studies have suggested that the lesion volume can vary depending on the magnetic field strength (48), suggesting that standardized imaging protocols are warranted. Although the Fazekas classification is simple and widely applicable across facilities, quantitative assessment using image analysis software may help reduce interobserver variability. As this study did not perform mediation analysis, determining the directionality of the associations among frailty, sarcopenia, and PVH was not possible. Furthermore, given that the social and economic backgrounds of the participants were not accounted for, the interpretation of the results should be approached with caution. In this study, sarcopenia was assessed using in-hospital data obtained after the onset of stroke, and the patients’ pre-stroke nutritional status could not be evaluated. Recent studies have suggested the utility of temporal muscle thickness (TMT) as a nutritional indicator in older adults (49). Incorporating TMT measurements may provide more objective data for evaluating nutritional status. Selection bias may have been present. The absence of a control group without AIS constitutes a limitation. Considering that the study population encompassed patients with AIS, future studies involving the general population are necessary.

In conclusion, PVH may have a greater impact on frailty and sarcopenia than DWMH, possibly due to differences in their underlying pathophysiological mechanisms and anatomical distribution.

## Data Availability

The raw data supporting the conclusions of this article will be made available by the authors, without undue reservation.
