# Supplementary material for: Associations of white matter hyperintensity with frailty, sarcopenia, and nutritional risk: an analysis of an acute ischemic stroke patient database
Source: Front Med (Lausanne). 2025 Oct 15;12:1631923. doi: 10.3389/fmed.2025.1631923 (PMC12568660; doi:10.3389/fmed.2025.1631923)
Supplement: Supplementary file 1 [file Table_1.docx]

Supplementary Material

# Supplementary Table 1. Univariate analysis of the presence or absence of frailty in patients with acute ischemic stroke

|  | Non–frailty (n = 70) | Frailty (n = 26) | P–value |
| --- | --- | --- | --- |
| Age (years) | 77.5 (60–95) | 79.5 (60–99) | 0.022* |
| Sex (male) | 37 (52.9%) | 21 (80.8%) | 0.011* |
| Height (cm) | 159.6 (135–180) | 160 (150–175) | 0.127 |
| Body weight (kg) | 59.5 (34–81.5) | 59.45 (44.8–82.3) | 0.951 |
| BMI (kg/m^2^) | 23.7 (14.3–33.8) | 22.2 (18.6–29.2) | 0.166 |
| DWMH grade | 2 (0–3) | 2 (0–3) | 0.342 |
| PVH grade | 1 (0–3) | 2 (1–3) | 0.003* |
| Grip power (kg) | 22 (2–45) | 20 (2–30) | 0.030* |
| Muscle mass (kg/m^2^) | 6.4 (4.2–8.9) | 6.5 (4.5–10.5) | 0.841 |
| Sarcopenia | 24 (34.8%) | 18 (72%) | 0.001 |
| NIHSS | 1 (0–18) | 3 (0–15) | 0.008* |
| Drinking history | 25 (35.7%) | 8 (30.8%) | 0.421 |
| Smoking history | 33 (47.1%) | 13 (50%) | 0.492 |
| Hypertension | 45 (64.3%) | 15 (57.7%) | 0.358 |
| Diabetes mellitus | 19 (27.1%) | 8 (30.8%) | 0.455 |
| Dyslipidemia | 28 (40%) | 9 (34.6%) | 0.406 |
| Albumin (g/dL) | 4.1 (2.7–5.0) | 3.95 (3.0–5.0) | 0.354 |
| Creatinine (mg/dL) | 0.84 (0.49–6.01) | 0.92 (0.55–9.1) | 0.055 |
| CRP (mg/dL) | 0.12 (0.1–10.02) | 0.14 (0.1–2.99) | 0.488 |
| HbA1c (%) | 6.0 (5.1–10.3) | 5.9 (5.4–8.6) | 0.511 |
| GNRI | 104.95 (76.4–129.5) | 102.05 (80.4–124.8) | 0.133 |
| GNRI nutritional risk | 1 (1–4) | 1 (1–4) | 0.238 |
| Pneumonia | 2 (2.9%) | 4 (15.4%) | 0.044* |
| UTI | 9 (12.9%) | 4 (15.4%) | 0.490 |
| Length of hospital stay (days) | 16.5 (5–68) | 21 (10–46) | 0.024* |
| mRS at discharge | 1.5 (0–5) | 3 (0–5) | 0.010* |

The numbers indicate the number of cases (%) or the median (minimum–maximum). *P-value < 0.05; Abbreviations: BMI, body mass index; CRP, C-reactive protein; DWMH, deep white matter hyperintensity; GNRI, Geriatric Nutritional Risk Index; HbA1c, hemoglobin A1c; mRS, modified Rankin Scale; NIHSS, National Institutes of Health Stroke Scale; PVH, periventricular hyperintensity; UTI, urinary tract infection

# Supplementary Table 2. Univariate analysis of the presence or absence of sarcopenia in patients with acute ischemic stroke

|  | Non–sarcopenia (n = 53) | Sarcopenia (n = 47) | P–value |
| --- | --- | --- | --- |
| Age (years) | 77 (60–95) | 81 (61–99) | <0.001* |
| Sex (male) | 33 (62.3%) | 29 (61.7%) | 0.559 |
| Height (cm) | 162 (138.7–180) | 160 (135–175) | 0.116 |
| Body weight (kg) | 63 (42.9–82.3) | 53.1 (34–72.3) | <0.001* |
| BMI (kg/m^2^) | 24.3 (19.2–33.8) | 21.3 (14.3–26.6) | <0.001* |
| DWMH grade | 2 (0–3) | 2 (0–3) | 0.114 |
| PVH grade | 1 (0–3) | 1 (1–3) | 0.003* |
| Grip power (kg) | 27 (3–45) | 15 (2–27) | <0.001* |
| Muscle mass (kg/m^2^) | 7.2 (5.1–10.5) | 5.6 (4.2–6.9) | <0.001* |
| NIHSS | 1 (0–18) | 3 (0–19) | 0.001* |
| Drinking history | 21 (39.6%) | 14 (29.8%) | 0.207 |
| Smoking history | 28 (52.8%) | 22 (46.8%) | 0.344 |
| Hypertension | 33 (62.3%) | 28 (59.6%) | 0.472 |
| Diabetes mellitus | 17 (32.1%) | 12 (25.5%) | 0.310 |
| Dyslipidemia | 21 (39.6%) | 17 (36.2%) | 0.441 |
| Albumin (g/dL) | 4.2 (3.3–5.0) | 3.9 (2.7–5.0) | 0.002* |
| Creatinine (mg/dL) | 0.86 (0.49–9.1) | 0.86 (0.49–6.01) | 0.714 |
| CRP (mg/dL) | 0.12 (0.1–3.0) | 0.13 (0.1–10.0) | 0.354 |
| HbA1c (%) | 6.0 (5.1–10.3) | 5.9 (5–8.6) | 0.487 |
| GNRI | 109.1 (93.3–129.5) | 98.8 (76.4–124.8) | <0.001* |
| GNRI nutritional risk | 1 (1–2) | 1 (1–4) | <0.001* |
| Pneumonia | 2 (3.8%) | 7 (14.9%) | 0.055 |
| UTI | 6 (11.3%) | 9 (19.1%) | 0.208 |
| Length of hospital stay (days) | 16 (7–52) | 21 (5–68) | 0.010* |
| mRS at discharge | 1 (0–5) | 3 (0–6) | <0.001* |

The numbers indicate the number of cases (%) or the median (minimum–maximum). *P-value < 0.05; Abbreviations: BMI, body mass index; CRP, C-reactive protein; DWMH, deep white matter hyperintensity; GNRI, Geriatric Nutritional Risk Index; HbA1c, hemoglobin A1c; mRS, modified Rankin Scale; NIHSS, National Institutes of Health Stroke Scale; PVH, periventricular hyperintensity; UTI, urinary tract infection
